# Assessment of Sperm Apoptosis and Semen Quality in Infertile Men-Meta Analysis

**Published:** 2012-03-01

**Authors:** T Fathi Najafi, M Hejazi, F Feryal Esnaashari, E Sabaghiyan, S Hajibabakashani, Z Yadegari

**Affiliations:** 1 Department of Shahinfar Medical, Islamic Azad University, Mashhad Branch, Mashhad, Iran; 2 Department of foreign languages, Islamic Azad University, Mashhad Branch, Mashhad, Iran; 3 Ferdowsi University, Mashhad, Iran; 4 Department of Midwifery, Islamic Azad University, Mashhad Branch, Mashhad, Iran; 5 Department of Nursing and Midwifery, Bojnord University of Medical Sciences, Bojnord, Iran

**Keywords:** Apoptosis, Sperm parameters, Male infertility

Dear Editor,

Apoptosis is the programmed cell death that occurs because of the DNA fragmentation which is seen in the sperm of infertile men.[1] There are two different mechanisms:

Intrinsic (mitochondrial) pathway and extrinsic pathway which is done by cell receptors.[2] Apoptosis has a significant relation with infertility of men.[3] The more immature the testicle, the more apoptosis will happen.[4] Standard seminal parameters do not reveal abnormalities in up to 20% of subfertile males.[4] Since the results of sperm analysis shows an average prediction of a person’s infertility[4] and infertility by the cause of male factors is 30-40% of the infertile couples, clinical examinations and new methods in male infertility can indicate a more accurate prediction for the state and function of the sperm. Therefore, sperm apoptosis is a strong and beneficial index for the state of male fertility.[4]

An internet study by the use of the key words “sperm apoptosis” and “sperm quality” through the databases Medline and Scopus from 2003 to 2009 has been performed. This study was done on human population. We relied on only English studies and all the articles we chose were clinical surveys. Due to the above mentioned reasons, the number of articles decreased and we have done the meta-analysis on the basis of 9 articles.

We selected the original articles that were done on volunteer human population. These articles were in two groups, comparing infertile and fertile men. The number of subjects was about 125 people. We have not chosen the articles which were biased or had minimum level of bias. Among the 20 initial articles, only 9 of them had the criteria to be chosen.

The results were taken from random effects model that is more conservative than a fixed effect one. Heterogeneity of effect sizes was done according to Cochran’s Chi-Squared (Q-test) and I2 statistics.[5] Heterogeneity of effect sizes was dependent on the type of variables that were in apoptosis examination and sperm quality. The meaningful heterogeneity showed the total credibility was not by chance.[5]

I2 statistic was from 0 percent to 100 percent in range which shows that the total proportion of variables in the studies was not because of chance values of 25 percent, 50 percent and 75 percent corresponding to low, moderate and high heterogeneity.[5] The effect of small-study and low quality of the studies was done through cumulative meta-analysis and the analysis of the subdivisions. Meta regression was also performed to recognize the covariance of the level of the study and its heterogeneity.[5]

CMA statistical software was used for this meta-analysis. The results showed that there was a significant difference between normal motility conditions of sperm and apoptosis (p=0/00) and by increasing the apoptosis intensity, the sperm motility which has an important role in sperm fertility decreases. Apoptosis is also exhibited by spermatozoa in the human ejaculate. These markers appear in excess in sub-fertile men and functionally incompetent spermatozoa.[2][6] Aziz in 2007 has shown that during apoptosis, disorder in the tail of sperm is because of positive annex that makes the movement of the tail difficult.[7]

Meta-analysis articles about the sperm concentration and their relations with apoptosis showed that apoptosis affected the sperm concentration and this can affect the fertility (p=0.001). This relation was similar to other studies.[8][9][10] The meta-analysis results according to a random model showed no significant relationship between the volume of semen fluid and apoptosis (p=0.275). Apoptosis is a cellular programmed process that can be performed by biochemical and morphological modifications. In this process without causing inflammation, the cell dies by the macrophages.[6][8][9][10] Before the cell contents leak and damage the surrounding cells, therefore not only necrosis does not happen, but also because of the lack of inflammation reaction, the components of the dying cell will be swallowed and recycled by macrophages. None of these processes will cause change in the vo-lume of cells and consequently in the semen fluid. The volume of semen fluid is not an important index in fertility.[10] We came to the conclusion that the intensity of apoptosis has a relationship with the decrease of sperm quality.
